# Name that tune: Melodic recognition by songbirds

**DOI:** 10.3758/s13420-016-0237-y

**Published:** 2016-07-21

**Authors:** Christopher N. Templeton

**Affiliations:** 1 Department of Biology, Pacific University, Forest Grove, Oregon USA; 2 School of Biology, University of St Andrews, St Andrews, Scotland UK

**Keywords:** Auditory perception, Avian communication, Melody, Musical cognition

## Abstract

Recent findings have indicated that European starlings perceive overall spectral shape and use this, rather than absolute pitch or timbre, to generalize between similar melodic progressions. This finding highlights yet another parallel between human and avian vocal communication systems and has many biological implications.

Language is one of the most elaborate traits to emerge from evolutionary processes. It lets us communicate abstract concepts with seemingly unending complexity. It has long been argued that language sets us apart from other animals. Certainly no animals have yet been discovered with the level of sophistication of human language, but the more we study animal vocalizations, the more parallels we find.

Songbirds are famous for their intricate vocalizations, and like language, many bird songs must be learned. Song learning is rare in the animal world, with the majority of mammals (even our closest relatives the apes) developing their vocalizations without much environmental input. In contrast, thousands of bird species in three different orders learn their songs, and song learning has many parallels with language learning (Doupe & Kuhl, 1999). For example, songbirds have a specialized song-learning brain pathway, with particular nuclei dedicated to processing auditory information and integrating with the vocal production pathway. Songbirds also have an early sensitive phase in which social stimuli are critical for promoting learning, and they even go through an overproduction (i.e., “babbling”) phase during development. Thus, the production and learning of bird songs appear to converge with human language in many regards.

Some similarities also exist between humans and songbirds in terms of auditory perception (Dooling, 2004). Birds have similar abilities to discriminate between frequency patterns, and are even better than we are at discriminating small differences in temporal sound patterns. These similarities make the numerous observations showing that birds do not perceive melody in the same way that we do surprising.

If you were to hear “Twinkle, Twinkle, Little Star” on a piccolo or a tuba, the tune would still be immediately recognizable because the pitch intervals stay the same across renditions, despite the absolute pitch being transposed. This focus on relative pitch is present early in development and appears to be universal in musical cognition, yet a variety of studies have shown that this ability is not one of the many parallels shared by songbirds. Previous research has instead suggested that songbirds use absolute pitch in the auditory perception of melodic sequences. Bregman, Patel, and Gentner (2016) have challenged this assumption with recent experimental findings.

Bregman et al. (2016) conducted a nice series of laboratory experiments examining auditory perception and learning in European starlings (*Sturnus vulgaris*). Starlings have complex songs, with relatively large repertoires that can incorporate mimicked songs from other species (Catchpole & Slater, 2008), making them ideal test subjects. First, Bregman et al. trained starlings to recognize a series of four-tone sequences that ascended or descended in pitch. Where most previous research had employed spectrally simple playback stimuli (i.e., pure tones), those used in this study had relatively complex spectral envelopes. Using an operant training procedure, starlings were rewarded for choosing the correct type of sequence, and they had very high accuracy rates after training.

Once the birds were proficient at recognizing these sequences, the authors conducted a series of experiments to determine whether they would generalize this knowledge to manipulated playback sequences. First, Bregman et al. (2016) manipulated the pitch (fundamental frequency) of the sequence and found that even small variations in pitch reduced the starling’s performance on the task to chance levels. Next, they manipulated the timbre (the distinct character of a musical sound, due in part to the relative amplitudes of harmonic overtones). They played the same sequence of notes on a piano instead of a synthesizer and again found that birds failed to generalize melodic sequences that preserved pitch but varied timbre from the training sequence. These results indicate that the melody was no longer recognizable to the birds after either absolute pitch or timbre had been manipulated, suggesting that neither of these cues alone is sufficient for auditory perception.

Finally, the authors investigated whether starlings use the overall spectral envelope of auditory stimuli, instead of timbre or pitch, to generalize melody. In this experiment, they used a technique from speech science called *noise vocoding* to remove pitch cues from the training sequences while still maintaining the spectral shape. This manipulation reduced the success rate of the starlings slightly, but the birds still did better than chance, and their performance improved with further test trials. These results contrast with the greatly reduced (chance-level) performance on another piano treatment, which kept the absolute pitch of the training sequence constant but manipulated the timbre. Together, these experiments indicate that starlings use spectral shape, rather than absolute pitch or timbre, to generalize across auditory information, and they have several implications for the study of animal learning and behavior.

## Implication 1: Neuroscience

Songbird brains have neural circuits that fire only when they hear particular songs. These song-selective neurons can function in discriminating conspecifics from heteropecifics, or can be tuned to respond only to particular syllables (Doupe & Kuhl 1999). Song-selective neurons also occur in the vocal-learning pathway and likely facilitate auditory feedback during the song-learning process. Bregman et al.’s (2016) results suggest that some songbird neurons could be tuned to particular spectral envelopes. It is already known that some neurons are sensitive to the temporal structure of song, so it is plausible that other features employed during auditory perception—like the spectral envelope—could be neurally encoded and used in song discrimination or as feedback during learning.

## Implication 2: Behavioral ecology

A large literature has examined the adaptive function of bird songs. Songs are generally used to attract mates and repel rivals, and can contain information that receivers use to discriminate between species and among individuals, including mates, specific territorial neighbors, and kin (Catchpole & Slater, 2008). Great tits appear to be able to generalize the vocalizations of particular individuals to other novel songs in their repertoires on the basis of some unknown voice characteristics, though song sparrows tested in a similar experiment did not show this ability to generalize across songs from a given individual’s repertoire. Chickadee song perception seems to be largely focused on the intervals between the song elements themselves. Thus, better understanding of the auditory processing involved in discriminating between different songs could help shed light on the signal structure of different species’ vocalizations and the evolutionary processes leading to speciation.

## Implication 3: Language and music cognition

Are animal vocalizations more analogous to human language or music? Clearly they transmit important information, but many are also aesthetically appealing, at least to us, and certainly starling songs fall into this category. Some birds mimic human music—for example, Mozart had a pet starling that whistled the theme from one of his piano concertos (West & King, 1990), further blurring the lines. The finding that birds rely on spectral shape in their auditory perception, rather than timbre or absolute pitch, suggests a similarity to human language rather than music. Human speech recognition is relatively unaffected by pitch degradation, much like the results observed for starlings in response to noise-vocoded song. In contrast, music perception is highly affected by similar manipulations of pitch. Furthermore, that starlings do not seem to have independent precepts of timbre and pitch—fundamental concepts to our musical understanding—suggests an inherent difference in their auditory perception. Further inquiry into auditory-processing strategies could lead to a more detailed understanding of the evolution of language and musical competence.

## Implication 4: Complexity of nature

The Bregman et al. (2016) study was particularly successful for two reasons. First, it questioned previous research, daring to use a different approach to readdress the issue of pitch perception in songbirds. Second, the authors employed complex stimuli that more accurately portrayed the types of stimuli that animals might encounter in the wild. Simplifying nature is often necessary to better understand the mechanisms involved in perception, but it is also important to remember that these simplifications do not necessarily re-create the experiences that animals would face in the wild. This study shows that mimicking more complex stimuli, which likely better mimic the types of stimuli animals might face in the wild, provides new insights into their learning and behavior. Further research should keep these findings in mind when attempting to simulate natural processes under experimental conditions.

## References

[CR1] Bregman MR, Patel AD, Gentner TQ (2016). Songbirds use spectral shape, not pitch, for sound pattern recognition. Proceedings of the National Academy of Sciences.

[CR2] Doupe AJ, Kuhl PK (1999). Birdsong and human speech: Common themes and mechanisms. Annual Review of Neuroscience.

[CR3] Catchpole CK, Slater PJB (2008). Bird song: Biological themes and variations.

[CR4] Dooling R, Marler P, Slabbekoorn H (2004). Audition: Can birds hear everything they sing?. Nature’s music: The science of birdsong.

[CR5] West MJ, King AP (1990). Mozart’s starling. American Scientist.

